# Optical Fiber Sensors for Structural Monitoring in Power Transformers

**DOI:** 10.3390/s21186127

**Published:** 2021-09-13

**Authors:** Catarina S. Monteiro, António V. Rodrigues, Duarte Viveiros, Cassiano Linhares, Hélder Mendes, Susana O. Silva, Paulo V. S. Marques, Sérgio M. O. Tavares, Orlando Frazão

**Affiliations:** 1 Department of Engineering Physics, Faculty of Engineering, University of Porto, R. Dr. Roberto Frias, s/n, 4200-465 Porto, Portugal; catarina.s.monteiro@inesctec.pt (C.S.M.); anvr@inesctec.pt (A.V.R.); carlos.d.viveiros@inesctec.pt (D.V.); paulo.v.marques@inesctec.pt (P.V.S.M.); 2 Centre for Applied Photonics, Institute for Systems and Computer Engineering, Technology and Science (INESC TEC), Rua do Campo Alegre, 687, 4150-179 Porto, Portugal; sfsilva@inesctec.pt; 3 Department of Physics and Astronomy, Faculty of Science, University of Porto, Rua do Campo Alegre, 687, 4150-179 Porto, Portugal; 4 Efacec Energia, Máquinas e Equipamentos Eléctricos, S.A., Apartado 1018, 4466-952 Porto, Portugal; cassiano.linhares@efacec.com (C.L.); hmendes@efacec.com (H.M.); sergio.tavares@efacec.com (S.M.O.T.)

**Keywords:** optical fiber sensors, power transformers, early fault detection

## Abstract

Power transformers are central elements of power transmission systems and their deterioration can lead to system failures, causing major disruptions in service. Catastrophic failures can occur, posing major environmental hazards due to fires, explosions, or oil spillage. Early fault detection can be accomplished or estimated using electrical sensors or a chemical analysis of oil or gas samples. Conventional methods are incapable of real-time measurements with a low electrical noise due to time-consuming analyses or susceptibility to electromagnetic interference. Optical fiber sensors, passive elements that are immune to electromagnetic noise, are capable of structural monitoring by being enclosed in power transformers. In this work, optical fiber sensors embedded in 3D printed structures are studied for vibration monitoring. The fiber sensor is encapsulated between two pressboard spacers, simulating the conditions inside the power transformer, and characterized for vibrations with frequencies between 10 and 800 Hz, with a constant acceleration of 10 m/s^2^. Thermal aging and electrical tests are also accomplished, aiming to study the oil compatibility of the 3D printed structure. The results reported in this work suggest that structural monitoring in power transformers can be achieved using optical fiber sensors, prospecting real-time monitoring.

## 1. Introduction

Power transformers, devices that transfer power between circuits using electromagnetic induction, are key elements of power distribution networks. Power transformers are one of the most expensive components of the power distribution network. The sustainability of transformers is of utter importance for maintaining power grid integrity. Faults in transformers can result in oil spillage, fires, or extensive damage in other grid equipment, leading to power distribution disruptions [1].

Network transformers yield long service periods, with an average of 30 years, but system degradation can accelerate their end-of-life [2]. Ageing and deterioration caused by corrosion is one important cause of transformer degradation. A survey of 2011 revealed that around 24% of all installed units in Portugal were older than 30 years [3], increasing the need for the monitoring of the health of transformers. In addition to ageing, the increased number of nonlinear and variable loads caused by distributed energy resources aggravates the demand on these systems [4]. Distributed energy resources, in particular the extensive use of plug-in hybrid electric vehicles (PHEV), introduce atypical power flows, congestion, and difficulty with system stability [5].

The rising age of power transformers and the increasing demand for electricity have prompted the development of low-cost, real-time monitoring systems. Currently, health monitoring can be accomplished by chemical, electrical, or acoustic methods [1]. Chemical methods consist of analyzing an oil sample to determine oil deterioration. This can be quantified by determining the water content, acidity, or interfacial tension, for example. Although it is a very accurate method, it requires an oil sample to be extracted for testing. Electrical and acoustic methods can be performed using electrical sensors such as strain gauges or impedance spectroscopy [6]. The ability to sustain radio frequency interference in harsh environments with corrosive or caustic atmospheres increases the difficulty and price of the sensor systems. Recently, wireless sensor networks have been studied for health monitoring but have a limited usage for power grids due to high electromagnetic interference and cyber security concerns [7].

Optical fiber sensors can be a viable alternative to conventional monitoring methods and are capable of multiplexing, multi-parameter analyses. Moreover, optical fiber sensors are small-sized, immune to electromagnetic interference, and resistant to harsh environments with high temperatures or acidity. Several different configurations have been proposed for health monitoring such as interferometric sensors based on Mach–Zehnder, Michelson or Fabry–Perot interferometry [8,9], or fiber Bragg gratings (FBGs) [4,10]. FBG-based sensors are one of the most prevalent fiber-based monitoring systems applied in health monitoring. In a recent study by Raghavan et al. [4], a multiplexed network of vibration and temperature FBG-based sensors was installed in an oil-filled transformer to assess the health of the transformer. After a laboratory validation, three transformers with embedded sensors were field tested for a period of six months with promising results in partial discharge and structural monitoring.

In this work, a 2D vibration sensor based on FBGs is presented and characterized for health monitoring in power transformers. The sensor, composed of a square cantilever structure, is studied regarding frequency and acceleration both analytically and experimentally. The sensor is embedded in a system that simulates the power transformer environment, both in dry and oil-filled conditions. The material compatibility with oil was studied using electrical and thermal aging processes.

## 2. Materials and Methods

### 2.1. Sensor Configuration and Fabrication

Fiber Bragg gratings (FBGs) are microstructures comprising a periodic modulation of the refractive index of the optical fiber core [11]. The periodic refractive index modulation leads to small light reflections that combine coherently at a particular wavelength defined by the spacing of the modulation. This wavelength is called the Bragg wavelength and it can be shifted by changing the modulation spacing by inducing strain, temperature variations, or other physical parameters on the fiber.

**Figure 1:**
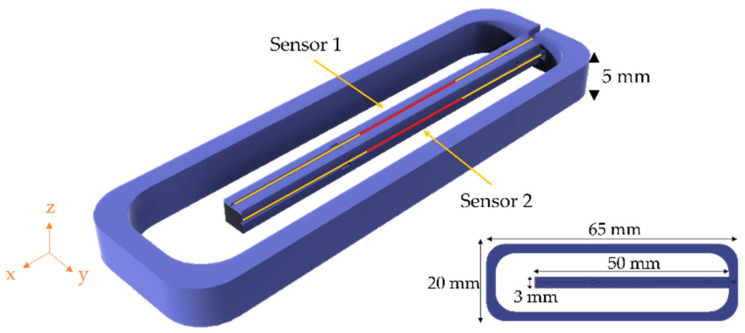
Schematic view of the designed cantilever structure with a square cross-section and the respective fiber position. In the inset, the relevant dimensions are annotated. The line in yellow represents the optical fiber and the red represents the position of the FBGs.

Vibration sensing with FBGs can be achieved by measuring the strain in the fiber induced by external vibrations [12]. FBGs can be embedded in structures that increase the mechanical response of the overall system, enhancing the sensor sensitivity [13,14,15]. In this work, two FBGs fabricated through femtosecond laser inscription, with a resonant wavelength of 1550 nm and a full width half maximum (FWHM) of around 1 nm and a high reflectivity, were embedded in a cantilever structure capable of being encapsulated in the pressboard spacers installed inside an oil-filled transformer. The two FBGs (named Sensors 1 and 2; Figure 1) were fixed near the neutral axis of the corresponding cantilever face, reducing the cross-sensitivity to vibration or curvature applied in other axes. The FBGs were fixed using an epoxy resin in the regions marked red in Figure 1.

The structure, presented in Figure 1, was fabricated using fusing deposition modeling, a 3D printing method, with a resolution of 90 µm. The material of the structure was acrylonitrile butadiene styrene (ABS), which exhibits a glass transition temperature of 106.4 °C. The dimensions of the structure were limited by the available volume in the pressboard spacers, with a 20 mm width, a 65 mm length, and a 5 mm height.

The undamped natural vibration frequencies can be calculated by the following equation [16]:

(1) $$f_{n}=\frac{\alpha_{n}^{2}}{2\pi }\sqrt{\frac{EI}{\rho AL^{4}\left(1-\nu^{2}\right)}}$$

where $\alpha_{n}$ are the solutions of the characteristic equation, E is Young’s modulus of the cantilever material, I is the moment of inertia, ρ is the material density, ν is Poisson’s ratio, and A and L are the area and length of the cantilever, respectively. Considering the first natural frequency, the solution of the characteristic equation, $\alpha_{1},$ is given by 1.875. For a square cantilever, the moment of inertia is given by $A^{2}/12$. Considering a Young’s modulus of 1.71 GPa, a density of 1.179 g/cm^3^, and a Poisson’s ratio of 0.375, typical values for ABS [17], the first natural frequency is around 251.8 Hz.

After printing the cantilever structure, the optical fibers were attached to the structure using an epoxy resin in the respective grooves (see Figure 1). The grooves were designed at the center of the respective section to minimize the vibration sensitivity applied at different axes.

### 2.2. Sensor Characterization

The cantilever sensor structure was characterized in terms of the acceleration amplitude and frequency by using an accelerometer (Model 301A11, PCB Piezoelectronics, New York, NY, USA) with a function generator (Model 58503A, Hewlett Packard, Palo Alto, CA, USA) and a voltage calibrator (Model 5790A, Fluke, Everett, WA, USA), respectively. It is important to mention that all tests were done in a temperature-controlled room to ensure that temperature fluctuations did not affect the measurements. Three characterizations were performed for the y and z axis, corresponding with the positions of the two FBGs. First, the cantilever sensor was directly placed on the accelerometer to characterize the printed structure. It was then embedded in a cut-out pressboard spacer and placed between two spacers and installed inside a tight box that simulated the conditions of the dry power transformer. This test was done to determine if the simulated conditions affected the structural response. The final characterization was performed by filling the box with mineral oil (Hyvolt I, Ergon International, Waterloo, Belgium) to study the influence of the presence of oil on the response of the sensing structure.

The optical signal was attained using the interrogation system BraggSCOPE (FS26, HBM Fibersensing, Maia, Portugal). This interrogation system is capable of simultaneously acquiring the reflected signal in two channels, with a maximum acquisition rate of 10,000 points per second and a dynamic resolution of 2.5 × 10^−3^ pm.

## 3. Experimental Results

### 3.1. Structural Analysis

**Figure 2:**
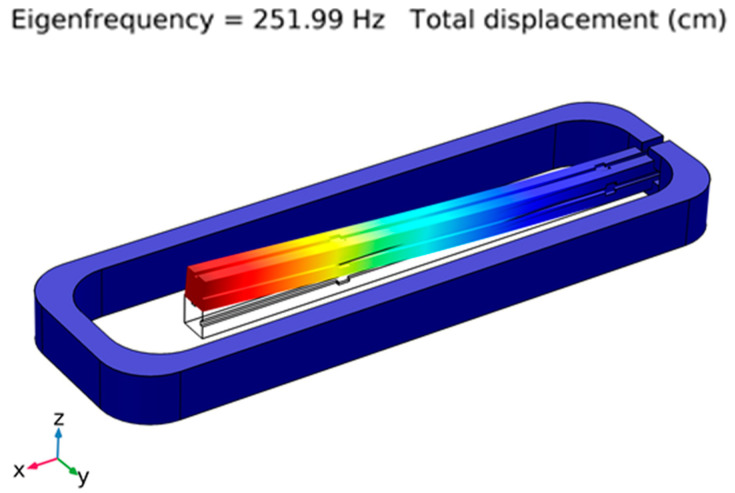
First eigenmode of the printed structure, determined using COMSOL Multiphysics^®^.

The mechanical properties of the printed structure were studied both analytically and experimentally. The analytical structural analysis was performed with COMSOL Multiphysics^®^ (COMSOL Inc., Stockholm, Sweden) using the solid mechanics module. The natural frequency modes were determined using the model of the printed structure, attaining a first natural frequency of 252 Hz and the vibration mode shown in Figure 2.

**Figure 3:**
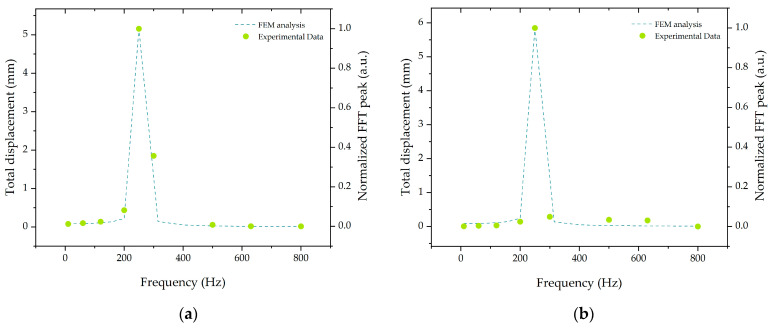
Cantilever displacement calculated by the FEM analysis in terms of applied frequency and experimental results for the normalized FFT peak for the (**a**) vertical and (**b**) horizontal direction.

The frequency-dependent deflection of the structure was studied by applying a 10 N load at the cantilever free edge with a harmonic perturbation with frequencies between 10 and 800 Hz. The maximum displacement of the cantilever was calculated and compared with the experimental results for the z-direction, as presented in Figure 3a. The experimental data were attained by applying a sinusoidal acceleration with a constant 10 m/s^2^ amplitude and a frequency in the range of 10 and 800 Hz. The experimental data were in accordance with those calculated analytically where a maximum fast Fourier transform (FFT) peak value at 250 Hz was attained near the eigenfrequency value and as expected. The same procedure was followed for the y-direction, attaining similar results as presented in Figure 3b. The normalized FFT peaks were determined by calculating the fast Fourier transform of the temporal signal and analyzing the peak with a higher amplitude. The results were then normalized to the highest value.

**Figure 4:**
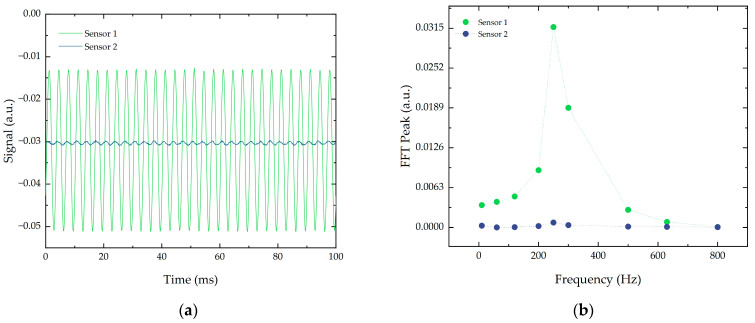
Output signal of the two sensors for a vertical vibration with an acceleration of 10 m/s^2^: (**a**) temporal signal and (**b**) corresponding FFT signal.

Another important factor is the sensitivity of the FBGs to vibrations applied in different directions because a small cross-sensitivity grants the directionality of the measurements. This means that, in addition to the frequency and the corresponding acceleration, the direction of the vibration can be inferred from the signals provided by the sensors. To study this cross-sensitivity, a vibration in the vertical direction was applied and the output signals from Sensors 1 and 2 were compared. The results, shown in Figure 4, indicate that the structure was capable of directionality in the measurements as the signal amplitude and the corresponding FFT peak were significantly smaller in Sensor 2, which, in turn, was mostly sensitive to the vibrations applied to the horizontal direction.

**Figure 5:**
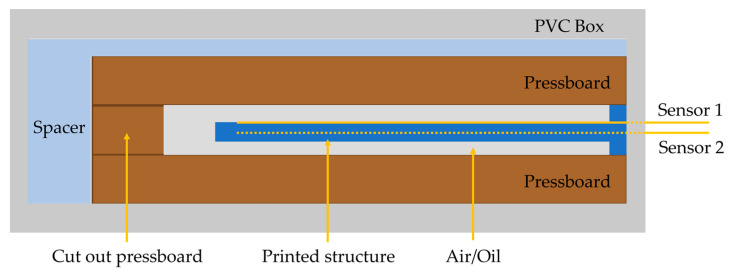
Schematic diagram of the simulated environment.

To simulate the conditions of a dry and oil-filled power transformer, the structure was embedded in a cut-out pressboard spacer and then enclosed between two other spacers. The replica of the dry power transformer was placed inside a PVC box to contain the system, as presented in Figure 5. A spacer was added to perfectly fit the pressboard assembly to the box, preventing additional structural vibrations.

### 3.2. Amplitude Characterization

**Figure 6:**
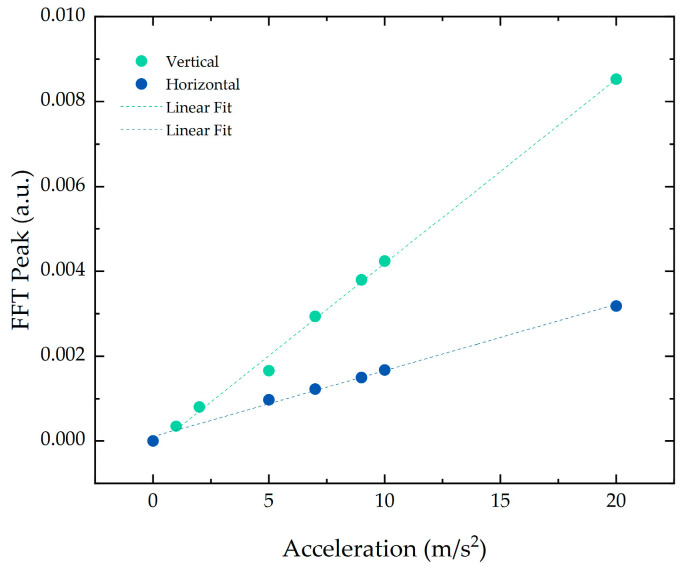
Signal peak value at 100 Hz for the different accelerations applied directly to the structure for the (green) vertical and (blue) horizontal direction.

The amplitude characterization was carried out by varying the acceleration of the applied force between 1 and 20 m/s^2^ with a fixed 100 Hz frequency, the value at which higher vibration levels due to the magnetostriction effect are expected [18]. The temporal signal was acquired at a frequency of 10 kHz and the respective FFT was calculated. The FFT peak value at a frequency of 100 Hz was determined for the applied accelerations, as shown in Figure 6. A linear response was attained in the two directions, exhibiting an r-squared of 0.995. The difference in slope may be due to slight variations between the position of the FBGs in the cantilever or due to a small sensitivity fluctuation between the reading channels of the interrogation system.

**Figure 7:**
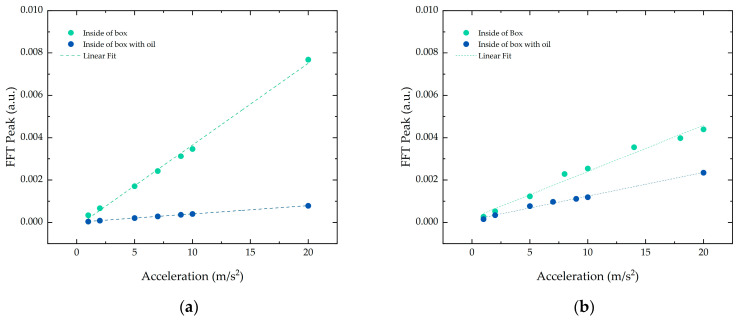
Signal peak value at 100 Hz for accelerations applied to the structure inside the simulation environment before and after adding the oil in the (**a**) vertical and (**b**) horizontal direction.

The inclusion of mineral oil in the simulated environment was also studied, as shown in Figure 7. The oil, which acted as a damper, contributed to a decrease in the FFT peak amplitude of 89% in the vertical direction and 46.5% in the horizontal direction. Nonetheless, the linear behavior of the FFT peak with the increase in acceleration was preserved under these conditions in both directions.

### 3.3. Frequency Characterization

The frequency characterization was performed by applying sinusoidal forces in the vertical and horizontal axes with a constant 10 m/s^2^ acceleration and a frequency varying between 10 and 800 Hz. It was possible to observe that, by enclosing the structure inside the simulated environment, the first natural frequency shifted toward higher values in both directions. This implied that the embedding structure of the sensor was affected by the presence of the pressboards. Moreover, a natural frequency decrease was observed in the presence of oil in the simulated environment. The resonant frequency of a rectangular cantilever beam immersed in a viscous fluid is given by [19]:

(2) $$f_{fluid}=f_{vacuum}{\left(1+\frac{\pi \rho_{fluid}b}{4\rho_{c}h}\right)}^{-1/2}$$

where $f_{vacuum}$ and $f_{fluid}$ are the resonant frequencies in the vacuum and in the fluid, b and h are the width and thickness of the cantilever, and $\rho_{c}$ and $\rho_{fluid}$ are the density of the cantilever material and fluid, respectively. Considering a resonant frequency in a vacuum of 252 Hz and a fluid density of 0.895 g/mL, the calculated resonant frequency of the immersed beam reduced to approximately 199 Hz, which was in accordance with the experimental results. The attained resonant frequencies under the different test conditions are summarized in Table 1.

### 3.4. Mineral Oil Compatibility

To study the viability of directly installed ABS-based structures in oil-cooled power transformers, electrical testing and thermal aging tests were performed on ABS samples. Considering that the sensor is designed for applications inside the transformer, it is crucial that the structure does not deteriorate the insulation properties of the mineral oil, in particular its dielectric strength. To study the electrical compatibility of the ABS structure with the oil, the dielectric strength of one ABS sample with dimensions 10 × 5 × 0.6 cm was determined. The sample was placed on an oil sample (Ergon International Hyvolt I) and attained a 91.5 kV/mm dielectric strength.

The typical working temperature of oil-cooled transformers is between 60 and 70 °C but can achieve temperatures as high as 100 °C [20]. Temperature increases can be caused by contaminants, increased water content in the oil, or oil aging and can lead to the failure of the equipment. The degradation of the ABS structure can also induce oil aging and, therefore, it is important to study the material stability under working temperatures. Aiming to study the oil aging induced by ABS degradation, two oil samples with a volume of 1 L were placed in an oven at 100 °C for 164 h. In one oil sample, a 30 g ABS piece was added and the other oil sample served as a blank test for comparison. After 164 h, the mineral oil with the ABS sample exhibited color variations, indicating the presence of contaminants. Regarding the acid number, the determined acidity of the oil was 0.013 mg KOH/g, lower than in the blank sample (0.024 mg KOH/g). Both were in the acceptable acidity range [21]. The inclusion of ABS also affected the breakdown voltage, the minimum voltage at which a dielectric material starts exhibiting conducting properties. For the sample containing ABS, the breakdown voltage was 75.1 kV and for the blank sample it was 70.7 kV.

The electrical and thermal aging tests indicated that ABS was compatible with the mineral oil. However, for high temperatures, ABS deforms as its softening temperature is approximately 100 °C [22], leading to measurement inaccuracy or, in extreme cases, to sensor faults. Therefore, for real applications, materials with higher softening points should be studied.

## 4. Conclusions

In conclusion, a vibration sensor based on a 3D printed square cantilever beam was presented for two-dimensional vibration sensing. The properties of the cantilever, such as the resonant modes and frequency, were studied both analytically and experimentally for frequencies up to 800 Hz. Furthermore, the potential application of this structure to oil-cooled power transformers was studied by embedding the cantilever structure on a cut-out pressboard to simulate the conditions inside a dry and oil-filled transformer. The experimental results showed that the sensor exhibited a linear response to accelerations between 1 and 20 m/s^2^ under all experimental conditions. The effects of the mineral oil on the response of the sensor were also studied, concluding that the oil acted as a damper system, decreasing the cantilever deflection. In addition, the inclusion of oil led to a decrease in the resonant frequencies as expected.

The material compatibility with the mineral oil was studied by performing electrical and thermal aging of ABS samples. The tests indicated that this material was compatible with the mineral oil typically found on power transformers; however, at higher temperatures, ABS deformed, leading to inaccuracy or faults in the sensor.

This study provides the basis for the development of a vibration sensor capable of detecting the acceleration and direction of vibrations. More research should address the temperature-dependent performance of the cantilever. Further studies should aim to find a material compatible with mineral oil and higher temperatures.

## Figures and Tables

**Table 1:** Resonant frequency in the vertical and horizontal direction for the three test conditions.

Test Conditions	Resonant Frequency (Hz)	
	Vertical	Horizontal
Bare structure	250	250
Structure enclosed in the box	300	300
Structure enclosed in the box with oil	200	200

## Data Availability

Not applicable.
